# 

**Published:** 1821-03-01

**Authors:** 


### BIBLIOGRAPHICAL RECORD.

In return for the favour of works transmitted to us by authors or publishers, we shall give the *full title pages,* so as to form a complete record of the works so received, the advantages of which, to the authors and publishers, are sufficiently obvious. Such gentlemen, however, as do not wish to have their works inserted in this list, will please to mark on the corner of the book or packet, the word "*private.*" The omission of this will be considered as permission for the insertion on the list.

### *Books received for Review since Dec. 1, 1820.*

1. The Pharmacopœia of the Royal College of Physicians of London, 1809, *literally* translated, and the chemical decompositions annexed. By George Fred. Collier, Surgeon, Licentiate of Apothecaries' Hall, Private Lecturer on Chemistry, &c. One vol. 8vo, pp. 231. London, 1821.

☞ *The title page sufficiently expresses the nature of this publication, which is designed for gentlemen preparatory to their passing at Apothecaries' Hall. The translation being literal, is a great advantage to them; and the chemical decompositions are asked at the Hall.*

---

2. A Statement of Facts, tending to establish an estimate of the true value and present state of Vaccination. By Sir Gilbert Blane, Bart. &c. &c. from the tenth volume of the Medico-Chirurgical Transactions, *with additions*. London, pp. 18. Nov. 1820.

☞ *See page* 718.

---

3. A Dissertation on the Disorder of Death; or that state of the frame under the signs of death called Suspended Animation; to which remedies have been sometimes successfully applied, as in other disorders, &c. &c. &c. By the Reverend Walter Whiter, Rector of Hardingham, Norfolk, and late Fellow of Clare Hall, Cambridge. One vol. 8vo, pp. 480. London, 1819.

☞ *See page* 687.

---

4. Medical, Geographical, and Agricultural Report of a Committee appointed by the Madras Government to enquire into the Causes of the Epidemic Fever which prevailed in the Provinces of Coimbatore, &c. &c. &c. By Drs. Ainslie, Smith, and Christie. One vol. 8vo, pp. 170, with a plate. London, 1816.

☞ *We beg to inform the publishers of this interesting work, who have forwarded us a copy, that in the monthly series of the Medico-Chirurgical Journal the work was amply reviewed and analysed—consequently we cannot enter again on its consideration.*

---

5. Pharmacopœa Pauperum, quam in usum Noscomii Regalis Metropolitani ad Morbos Puerorum debellandos, sub auspiciis Regis Augusti Georgii IV. Anno salutis MDCCCXX. fundati, et perenni Memoriæ Altissimæ Principissa Walliarum Carolotta dicati, Medeci et Chirurgi Statuerunt Londini, 1820. Octavo, pp. 32. By Dr. Granville.

☞ "*The produce, says the Author, of the sale of the present Pharmacopœia, as well as of the Synopsis of the Diseases of Children, recently published by Dr. Granville, is intended for the benefit of the Infirmary.*"

6. Recherches Anatomico-Pathologiques sur l'Encephale et ses dependances. Par F. LALLEMAND, Professeur de Clinique Chirurgicale a la Faculté de Medecine de Montpellier, &c. &c. One vol. 8vo, pp. 100. Paris, 1820. (*Lettre premiere.*)

---

7. Cases illustrative of the Treatment of Obstructions in the Urethra, &c. by the new Instrument, THE DILATOR; with further directions to facilitate its general adoption. Also a case of extraction of stone from the male bladder, without cutting, by the Dilator; with an account of improvements of the method of dissolving stone by injection, and of the common operations of Lithotomy. By JAMES ARNOTT, Member of the Royal College of Surgeons, London. One vol. 8vo, pp. 119. London, 1821. *With a plate.*

☞ *See page* 743.

---

☞ *The following Italian Works have been transmitted to us for review by our friend and correspondent, Mr. Todd, af Pisa, in Italy.*

8. Della Nuova Dottrina Italiana. Prolusione alle lezione di Clenica Medica Nella P. Universita di Bologna per l'anno Scolastico 1816-1817. Del Professore GIACOMO TOMMASINI. One vol. 8vo, pp. 98. Firenze, 1817.

---

9. Prospetto de Resultamenti ottenuti Nella Clinica Medica. Della Pontificia Universita di Bologna nel corso di un triennio scolastico, 1819-1820. Del Professore GIACOMO TOMMASINI. Octavo, pp. 47. Pisa, 1829.

---

10. Lettere del Cavalier Professore ANTONIO SCARPA, Al Cav. Professore A. Vacca Berlinghieri, sulla legatura della Grosse Arterie degli arti e risporta alle Medesime del Cav. Professore A. VACCA BERLINGHIERI. Pisa, 1820.

---

11. Memoria Sopra l'allacciatura dell' Arterie del Dottore ANDREA VACCA BERLINGHIERI. Pisa, 1819.

---

12. Della Esofagotomia e di un Nuovo Metodo di Eseguirilla, Memoria di ANDREA VACCA BERLINGHIERI. Pisa, 1820.

---

13. (*From the Author.*) Recherches et Observations Physiologiques et Chirurgicales sur les anus contre Nature. 2*d Partie.* Par GILBERT BRESCHET, Docteur en Medecine, Chef des Travaux Anatomiques, &c. &c. a Paris. 102 pages of M.S. and five plates. Paris, 1821.

☞ *This very important manuscript we have transmitted to our respected cotemporary, "The Quarterly Journal of Foreign Medicine;" and when the whole is published, we shall present an analysis of the paper to our readers.*

14. Considerations Generales sur les Fistules et sur la formation d'un tissa accidentel dans leur trajet; suivies d'observations recueillies à la clinique de M. le Professeur Dupuytren, sur different especes de Maladies de ce genre et leur mode particulier de traitement. Par G. Breschet, &c. &c. &c. *(premier article)* pp. 16. 1821.

---

15. Dell' Infiammazione e della febre continua. Considerazioni Pathologico-Practiche. Di G. Tommasini, Professore di Clinica Medica Nella Universita' Di Bologna, &c. One vol. 8vo, pp. 272. Pisa, 1820.

☞ *We hope soon to give an account of this important Work.*

---

16. Dicours prononcé par M. Le Professeur Richerand, President de la Faculté de Medecine à Paris. Seance publique du 7 Nov. 1820. Quarto, pp. 20.

☞ "*Desormais rendue a son unité primitive, la medecine en France, offre a peine de legères traces de ces divisions facheuses qui firent si long temps la joie de ses detracteurs, et mirent obstacle à ses progres.*" *The professor deplores, however, the degraded state of the profession in his country, in consequence of the medical market being overstocked—partly arising from the* gratuitous *system of medical education. The rapid increase of population, he thinks, has driven a surplus of candidates into every liberal profession, but more particularly into the medical.* "*The hope of gaining a subsistence is the real cause,* de la multitude des medecins *et des abus qui naissent de cette facheuse multiplicité.*" *From this dire necessity, says M. Richerand, of procuring* something to eat *(le besoin de diner tous les jours) has sprung the trade of editing medical journals, in which beardless boys endeavour to procure subscribers by abusing their masters.* "*Cherchent a gagner quelques abonnés en injuriant leurs maitres.*" *P.* 11.

*The Professor lashes* "*les transactions honteuses entre les medecins qui ordonnent et les pharmaciens qui preparent les medicamens,*" *and the pretenders to secret remedies. But he laments to say that there are medical men who injure the profession, not from want, but from insatiable ambition.* "*It becomes,*" *says he,* "*more and more difficult, every day, to gain distinction in the first ranks of medicine, where a crowd of able and learned men are thronging every avenue to the temples of Fame and Fortune. Art is therefore called in to the assistance of Talent, and the most abominable machiavelian politics are made to violate all the laws of probity and honour, in subserviency to the thirst of fame, or the love of money.*" *The Professor compares these desperate, and almost maniacal struggles for distinction, to the furious efforts of our unfortunate countrymen in the black hole of Calcutta, while trampling over the dead and dying to get to the only chink which admitted light and air:—*"*foulant aux pieds les cadavres de leur compagnons, s'efforcaient d'atteindre l'unique soupirail par lequel l'air et la lumiere penetraint dans cet antre horrible!*"

*Such we hope is an exaggerated picture of the state to which the science of medicine has come on the Continent. For our own parts, we prefer those "divisions facheuses," which, in this country, has as yet secured some degree of respectability in our profession, to that homogeniety, but deterioration of character, too evidently conspicuous in the present system of things elsewhsre. It may be wise for us, however, to take a lesson from the state of professional affairs on the Continent, and prevent, if possible, a similar scene here.*

---

17. A History of the Epidemic Fever which prevailed in Bridlington and the Neighbourhood, in the years 1818 and 1819. By Humphrey Sandwith, Surgeon, Bridlington. Also, Observations in Medicine and Surgery, by Thomas Sandwith, Surgeon, Beverly. One vol. 8vo, pp. 320. London, 1821.

---

18. Recherches Anatomico-Physiologiques sur l'encephale et ses dependances. Par F. Lallemand, Professeur, &c. a Montpellier. *Seconde Lettre*, pages 222. Paris 1821. *Transmitted by M. Breschet.*

☞ *Both Letters in our next.*

---

19. Annals, historical and medical, during the first four years of the Universal Dispensary for Children, St. Andrew's Hill, Doctors' Commons, founded in 1816; for the sole purpose of affording prompt medical aid to the children of the necessitous poor, from the period of their birth to the age of twelve years, from all parts of the metropolis and its vicinity, &c. to which is added, a concise essay in elucidation of the rules and methods adopted at the Institution, on the bodily, management of children, &c. &c. Edited by John Bunnell Davis, M. D. of the Royal College of Physicians of London—Senior Physician to the Universal Dispensary, &c. One vol. 8vo, pp. 648. 1821.

---

20. Practical Observations in Midwifery; with a Selection of Cases. Part I. By John Ramsbotham, M. D. Lecturer on Midwifery at the London Hospital, and one of the Physician Accoucheurs to the Lying-in-Charity for delivering poor married women at their own habitations. One vol. 8vo, pp. 422. London, 1821.

---

21. Observations on the Climate of Penzance, and the District of the Land's End, in Cornwall; with an Appendix, containing meteorological tables, &c. &c. By John Forbes, M. D. Octavo, sewed, pages 64. London, 1821.

☞ *See page* 695.

---

22. History and method of cure of the various species of Palsy; being the first part of the second volume of a treatise on *Nervous Diseases.* By John Cooke, M. D. F. A. S. Fellow of the Royal

College of Physicians, and late Physician to the London Hospital. Octavo, pp. 215. London, 1821.

☞ *See page 725.*

---

23. Practical Observations on Chronic Affections of the Digestive Organs, and on Bilious and Nervous Disorders; being an attempt to combine with English practice some useful methods of cure employed on the Continent. Also, Remarks on Warm Mineral Baths, Mineral Waters in general, and on the Use and Abuse of the Cheltenham Waters. By John Thomas, M. D. many years resident physician at Toulouse, now practising physician at Cheltenham. Octavo, pp. 168. London, 1821.

---

24. A Dictionary of Chemistry, on the basis of Mr. Nicholson's; in which the principles of the science are investigated anew, and its applications to the Phenomena of Nature, Medicine, Mineralogy, Agriculture, and Manufactures, detailed. With an introductory dissertation containing instructions for converting the alphabetical arrangement into a systematic order of study. By Andrew Ure, M. D. Professor of the Andersonian Institution, Member of the Geological Society, &c. Royal octavo, double columns, price one guinea.

☞ *This appears to us to be a very valuable work, of which we hope to be able to give some account in our next.*

---

25. Opinion de M. Lefort, Medecin du Roi a la Martinique, sur la Non-contagion et Non-importation de la Fievre Jaune. Publiée avec des notes, par M. M. Sedilot. Octavo, pp. 24.

☞ *It is hardly necessary to state that this opinion of M. Lefort has long been the conviction of the greater number of experienced observers in this country.*

---

26. Engravings of diseased appearances, taken from drawings, with the symptoms, progress, and treatment of the cases. By John Harrison, Member of the Royal College of Surgeons, and Assistant Surgeon, First, or Grenadier Regiment of Foot Guards. Royal quarto, three coloured plates, with letter-press description. London, 1821.

☞ *This is the first fasciculus of a series of delineations of those diseases usually called venereal. The plates are very well executed, and the attempt appears highly deserving of public encouragement. In our next we shall give a more particular account of the present fasciculus.*

---

27. Revue Medicale No. VI. for January 1820; containing, among other varieties, an extensive review of Dr. Clark's Medical Notes on Climate; Dr. Bateman on Diseases of the Skin; the controversy between Dr. Philip and others, from this Journal, &c. &c.

28. A Treatise on the Epidemic Cholera of India. By JAMES BOYLE, Surgeon of H. M. S. Minden. Octavo, pp. 78. London, 1821.

---

29. A Treatise on Gun-Shot Wounds, on Injuries of Nerves, and on Wounds of the Extremities, requiring the different Operations of Amputation; in which the various methods of performing these operations are shewn, together with their after-treatment; and containing an account of the author's successful case of amputation at the *hip-joint, &c. &c.* with five explanatory plates. Being a record of the opinions and practice of the surgical department of the British Army, at the termination of the war in Spain, Portugal, and France, in 1814. *Second edition, considerably enlarged.* By G. J. GUTHRIE, Deputy Inspector of Hospitals during the Peninsular War; Surgeon to the Royal Westminster Dispensary for Diseases of the Eye; Consulting Surgeon to the Western Dispensary for Diseases of Women and Children, Lecturer on Surgery, &c. &c. &c. One closely printed volume, 8vo, pp. 540, five plates. London, 1821.

☞ *Of this highly valuable volume we hope to present a comprehensive account in our next number.*

---

30. Aperçu Philosophique sur la possibilité de Perfectionner l'Homme par les Modifications de son Organization. Par M. A. DESMOULINS, Docteur en Medecine, &c. Bruxelles, 8vo, sewed, pp. 28. *(From M. Breschet.)*

---

31. Notice sur l'Inflammation Aigue de la Substance Medullaire du Rachis. Par PINEL, Fils, D. M. P. Octavo, pp. 16. Paris, 1821. *(From M. Breschet.)*

---

32. Journal de Physiologie Experimentale. Par F. MAGENDIE, D. M. &c. &c. No. 1, Janvier, 1821. Octavo, pp. 96, with a plate.

☞ *This first No. of a Journal dedicated to a most interesting department of Medical Science, reached us too late for analytical notice this quarter. It contains, among other important articles, two memoirs on absorption by Magendie—one on the introduction of viscid liquids into the blood—experiments on hydrophobia by the same—on inflammation of the spinal marrow—experiments on veratrine—memoir on the structure of the lungs in man; on the varieties of this structure at different epochs; and on the origin of phthisis pulmonalis. By F. Magendie—Recherches Anatomiques et Chimiques sur un Hydrocephale Chronique, par M. Breschet, &c. &c. &c. We shall give a farther account of this interesting stranger in our next number. In the mean time we are much mistaken, if it will not be found one of the most important professional works which the periodical press of Paris pours forth with such profusion.*

In the Press, and speedily will be published in octavo, volume the first, of the *Principles* of *Medicine*, written entirely on the plan of the Bacconian Philosophy, to prove, that the only rational method of curing Disease, is to induce, by Medicine, an opposite or counteracting action, sufficiently powerful to expel the Disorder. By R. D. Hamilton, Medical Practitioner.

---

In the Press, and will shortly be published, The Principles of Forensic Medicine, explained, illustrated, and applied to British Practice. By J. G. Smith, M. D.

---

*To Parents and Guardians.*

The Editor of this Journal is acquainted with a Medical Gentleman in London, of talents and respectability, Surgeon to a public Institution, and in extensive private practice, who will admit a youth as an articled pupil or apprentice, who will have opportunities of prosecuting his studies with many and peculiar advantages, which can only be appreciated by medical men.

The Editor will give further information, and names of parties, if applied to.

---

Mr. Haden, of Sloane-street, is about to publish a Monthly Journal of Medicine, addressed principally to unprofessional persons;—not designed to teach the cure, but the causes and prevention of diseases.

---

L. Towne has in the Press, and speedily will be published, The Farmer and Grazier's Guide; containing a collection of valuable Recipes, for the most common and fatal Disorders to which Horses, Horned Cattle, and Sheep, are subject; both tried and approved of by many of the greatest farmers in the land.

---

*Additional Subscribers since last Quarter.*

---

Ayton, Mr. Robinson, Surgeon, Porchester, Hants
Asiatic Journal, Proprietors of, in exchange for the Medico-Chirurgical Review
Acton, Mr. Surgeon, Ludlow
Boyle, Mr. James, late Surgeon, H.M.S. Minden
British Review, Proprietors of, in exchange for the Medico-Chirurgical Review
Blennerhasset, J. H. Esq. Surg. H. C. ship, Lowther Castle
Burke, Dr. Pembroke
Bartlett, Mr. Surgeon, Great Bedwin

Benwell, Henry, Esq. Memb. Roy. Coll. Surgeons, London, and Surgeon to the Kent Dispensary
Baxton, Mr. Surgeon, Knighton
Billet, Thomas, Esq.
Channing, Dr. Professor of Midwifery and Medical Jurisprudence, Harvard University, New England, in exchange
Cosgreave, Dr. Peter, Surrey-street, Strand
Cox, Mr. Hon. Comp. Service
Crosley, Mr. Surgeon, (place not stated)
Church, Mr. Acton
Carrick, Mr. Surg. Kennington
Coates, Mr. Surgeon, Haydon-bridge, Hexham
Collins, John, Esq. Surgeon
Dempster, Mr. John, Assistant-Surgeon, Cape of Good Hope
Douglas, John, M.D. New South Wales
Dill, John, Esq. Surgeon, E.I.C. ship, Atlas
Dickinson, Mr. Wigmore-street
De Sanctis, Dr.
Davis, Dr. St. Kitts
Diac, Mr. James, A.M. Leek
Dillon, Charles, Esq.
Faulkner, Sir Arthur Brooke, M.D. Cheltenham
Freers, W. F. Esq. Surgeon, Stourbridge
Forster, Mr. Edward
Gairdner, Dr. Dumfermline
Golding, Benj. Esq. Surgeon, St. Martin's-lane
Goldie, Dr. George, York
George, H. Esq. Surgeon, Kensington
Gibson, Mr. Erasmus
Howship, John, Esq. George-street, Hanover-square
Husband, Mr. W. Member of the Royal Coll. of Surg. York
Houghton, Mr. Conduit-street
King, Mr. George, Member of the Royal Coll. of Surg. and Society of Apoth. London;—Harxest, Suffolk
Lamert, M. Esq. Surg. 1st. Reg. Vet. Bat. Chatham
Latham, Dr. P. M. Fellow of the Roy. Coll. of Physicians, Gower-street
Lucas, Dr. Brecknock
Lloyd, William, M.D. Memb. Roy. Coll. Surg. and Assistant-Surgeon, Chatham
M'Carthy, Dr. Alexander Skibbereen, Cork
Miles, Mr. Surgeon, Throgmorton-street
Magendie, Dr. Paris, in exchange for Le Journal de Physiologie
Noble, Mr. Martin, Memb. of the Roy. Coll. of Surg. Shipton, Birmingham
Omodei, Signor, Milan
Parson, Mr. C. A. Godalmin
Percy, Dr. H. G. to Cape of Good Hope, with His Excellency, Sir R. S. Donkin
Palin, Dr. Newport, Shropshire
Ritchie, Mr. Surg. Sierra Leone
Rogerson, Mr. John, Apothecary, Blackburn, Yorkshire
Stone, Mr. Thomas
Sabine, Dr. Kington, Herefordshire
Stewart, Mr. Manchester
Scott, Mr. Surgeon, Easingwald
Todd, John T. Esq. Pisa
Tommasini Giacomo, Clinical Professor, University of Bologna
Vetch, John, M.D. F.R.S.E. Regent-street
Vidal, Mr. Surgeon, Dean-st. Soho
Wilson, W. Surgeon, Arbroath, R. N.
Wood, Mr. Surgeon, Middleton

### SUBSCRIBERS.

Abell, Dr. Brighton
Adams, Sir W. Albemarle-street
Addison, Dr. Hatton Garden
Addison, Mr. J. Surg. Burnham
Alcock, Mr. Surg. Piccadilly
Allen, Mr. J. Surg. Weymouth
Allinson, Mr. J. H. Surgeon, Woolwich
Alexander, Mr. John, Surgeon, Newbury.
Allan, Mr. J. Surg. Jermyn-st.
Anderson, Mr. W. Surg. R. N.
Anderson, Dr. R. N.
Anderson, Mr. Surg. Bouverie-st.
Andrews, Mr. Surg. Richmond
Ange, Mr. James, Fareham
Armstrong, Dr. John, Southampton-row
Arnott, Dr. Bedford-square
Ashford Medical Society, Kent
Ashwell, S. Esq.
Asiatic Journal, Proprietors of
Austin, Mr. John Haslemere, Surry
Ayres Mr. Mary-le-bone street
Ayton, Mr. Robinson, Surgeon, Porchester

B.

Bacon, Dr. Charles Edward, Guilford
Baillie, Dr. Mat. Cavendish-sq.
Bailey, Dr. Reading, Berks
Bailey, Mr. Harwich
Bailey, Mr. Surgeon, Blackburne
Bailey, Mr. Sam. Surgeon, Cape of Good Hope
Balantine, Mr. Middlesex Hosp.
Baron, Dr. John, Gloucester
Bartley, Dr. Assist. to the Forces
Barry and Co. Broad-street Buildings
Bath, Dr.
Bath, Jacob, Esq. Surgeon to the Forces
Barry, Dr. Clifton
Bartlett, Mr. Ipswich
Barton, Mr. Manchester
Bampfield, Mr. 31st Regiment
Bampfield, Mr. Bedford-street
Baynton, Mr. Bristol (since dead)
Barton, Francis, M. D. Whitby
Bartlett, P. Esq. Nottingham-place
Beaty, Dr. Physician of the Fleet
Bell, Mr. Newry
Bennett, Mr. Shifnal, Salop
Belcombe, Dr. H. S. Newcastle-under-line
Bean, Mr. Surg. Camberwell
Best, Mr. Tavistock-street
Benwell, Henry, Esq. Memb. Roy. Coll. Surgeons, London, and Surgeon to the Kent Dispensary
Bent, Mr. Diss.
Bell, Mr. Assist. Surg. R. N.
Bell, Mr. Alex. Surg. Dundee
Berryman, Dr. Penzance
Berry, Mr. Surg. Windsor
Belcombe, Dr. York
Bishop, Mr. Surg. London
Birckhead, Dr. L. Baltimore
Bisset, Mr. Charles E. Surgeon, Peckham
Blacklock, Mr. Surg. Dumfries
Blake, Mr. Chemist, Piccadilly
Blacket, Mr. Charles P. Park-st.
Blennerhasset, J. H. Esq. Company's Service.
Blood, Mr. Westminster Hosp.
Bowman Mr. D. Harley-street
Boylston Med. Society, America
Bourne, Mr. Surg. Cheadle
Boxwill, Mr. Surg. Ireland.
Boutflower, C. Esq. Surgeon to the Forces
Bowen, Dr. Caermarthen
Boulton, Thos. Esq. Thornbury

Bradley, O. Esq. Surgeon, Westmoreland Milit.
Braine, J. W. Esq. Oxford
Breschet, Dr. Paris
Bree, Dr. George-street, Hanover-square
Broadfoot, Dr. Corfu
Bruce, N. Esq. Sandhurst
Brigham, Mr. Manchester
Broughton, S. Esq. Falmouth
British Review, Proprietors of
Brera, Professor, Pavia
Bridger, Mr. H. C. Service
Brookes, B. Esq. Surg. Reading
Brookes, Mr. Surg. Wenlock
Burnett, Dr. Chichester
Burnaud, Mr. Chichester
Burchell, Mr. Hackney
Bushell, Mr. Russell-st. Covent Garden
Bullen, Mr. Surg. Ipswich
Bullen, Mr. George
Burrows, Dr. Gower-street
Burnidge, Mr. T. D. Worcester
Butini, Dr. Adolphus, Geneva
Buxton, J. Esq. Gatton Park, Ryegate
Buchanan, Mr. Surg. City Road
Burgoyne, Mr. Surg. H.C. Serv.
Byass, Mr. Surg. Arundel
Byass, Mr. Surg. Crookfield

C.

Caddy, Mr. Surg. Great Torrington
Callow, Mr. 20th. Lt Dragoons
Calley, Mr. Surg. East Essex Militia, Colchester
Campbell, Mr. Dougall, Picardy-place, Edinburgh
Canim, W. Esq. Nottingham
Candler, M. Grundesburgh
Carbutt, Dr. Manchester
Carden, Arthur, Esq. Templemore
Carmichael, R. Esq. Dublin
Carrol, William, Esq. Surgeon, Walworth-road
Carver, Walter, Esq. Surg. 18th Regiment
Cary, Mr. J.W. Surgeon, Trowbridge
Cashill, Dr. Hemphill
Chambers, Mr. Charles, Surg. Leamington
Channing, Dr. Professor, Harvard University
Chapman, Mr. Surg. Windsor
Chatham, Medical Society
Cheyne, John, M. D. Dublin
Child, Mr. Assistant Surgeon Madras Establishment
Chilvers, Samuel, Esq. New Burlington-street
Christie, Dr. Thos. Cheltenham
Church, W. H. Esq. East Acton
Clark, Dr. James, Rome
Clark, James, M. D. Dublin
Clark, Rev. Dr. John, Dublin
Clarke, Dr. J. Cuthbert, Smyrna
Clarke, T. Esq. Newman's Row
Clarke, Sir Arthur, Dublin
Clarke, Mr. Windmill-street
Clarke, J. Esq. Surgeon, Currie
Clements, Mr. Wadebridge, Cornwall
Clifford, Surgeon, Trim, Ireland
Clifton, Mr. London
Coates, I. Esq.
Cock, Mr. Easingwald
Cole, Dr. John, Surgeon to the Forces
Coley, J. M. Esq. Bridgenorth
Cooper, Mr. Portsea
Copland, Dr. Walworth-terrace
Corsan, John, Esq. Royal Navy
Corsham Medical Book Society, 2 copies
Cooke, Dr. John, Gower-street
Cooper, Astley, Esq. Spring-gardens
Colquhoun, Dr. Craven-street, Strand
Cornish, Dr. James, Falmouth
Coyne, Phineas, Esq. Welbeck-street
Cosgrave, Mr. Batavia
Cosgreave, Mr. Peter, Surry-st. Strand
Cockburn, Dr. Mark Dunse

Cox, Mr. Hon. Comp. Service
Cowen, Mr. Ireland, (town not stated)
Crampton, Philip, Esq. Surgeon General, Dublin
Crane, William, M.D. Boston
Crawford, Dr. Bath
Creasy, Mr. Surg. Eden Bridge, Surrey
Creighton, Mr. Andrew, R.N.
Crichton, Dr. West Indies
Crucifix, Mr. Surg. Bouverie-st.
Cuming, Dr. Dublin
Cunningham, Mr. Plymouth
Curry, Dr. (since dead)
Curtis, Mr. Lisson-street, New-road
Curtis, J. H. Esq. Soho-square
Curtis, John, M. D. Cowley
Cuthbert, Mr. Suffolk

D.

Daniels, Mr. Princes-street
Darrack, Mr. Philadelphia
Davidson, Mr. Edinburgh
Davidson, Mr. Alexander
Davidson, Dr. Nottinghamshire
Davidson, Dr. J. M. Hanley, Staffordshire
Davie, Mr. Ipswich
Davis, Dr. John Bunnell
Davies, Mr. W. Orchard-street
Davidson, Dr. J. M. Hanley
Davies, Mr. W. L. Surg. Poland-street
Davies, Mr. Conduit-street
Davis, Dr. George-street
Dawson, Ensign, St. Kitts, West Indies
Dean, Mr. Surg. Standress
Delph, Mr. Surg. Edmonton
Dempster, Mr. Assist.-staff-surg. Harwich
Dempster, Dr. (place not mentioned)
Denmark, Dr.
Denicke, Dr. Isle of Wight
Debishire, Mr. John, Cheadle
Derinzy, Dr.
Dewar, Dr. Edinburgh
Dickson, Dr. Clifton
Dillon, Mr. Surg. Lambeth-terrace
Dickenson, Mr. Milcham
Dickenson, Mr. Sloane-street
Dickinson, Mr. Wigmore-street
Dobie, Mr. Exmouth-street
Dobson, Dr. Chatham Division of Marines
Douglas, J. M.D. Dublin
Downe, Dr. J. S. Florence
Drew, Mr. James, Gower-street
Drake, Richard, Esq. York
Draper, Mr. Surgeon, Burford, Oxfordshire
Ducamp, M. Docteur en Medecine à Paris
Dugdale, Mr. Blackburn, Lancashire
Duncan, Mr. Torrington-place
Dwyer Dr. John, Ceylon

E.

Eberle, Dr. John, Philadelphia
Editor of New Monthly Magazine, in exchange
Ellerby, Mr. Canon-street
Elliot, J. Esq. Surg. to the Forces
Elmslie, Mr. Berners-street
Emery, Dr. Physician to the Forces
Emerson, Dr. A. L. Brompton
Evans, Surgeon, Kilkenny
Evans, Mr. William, Surg. R.N.
Evans, Dr.

F.

Farnder, Joseph, Esq. Surgeon, Canada
Fairchild, Mr. Surgeon
Fallofield, Mr. Albemarle-street
Fearn, Mr. T. Huntsville, State of Alabama
Fewster, Mr. Thomas, Surgeon, Thornbury
Felix, Mr. Southsea
Fiddes, Mr. 85th Regiment
Filson, Mr. Robert, Madras
Fitzpatrick, Dr. Woolwich
Flanagan, J. B. Esq. Surg. 9th Regt. Vet. Battalion
Fletcher, Mr. Arundel
Foot, Mr. Jessé, Jamaica

Foot, Mr. George Forester, Jamaica
Forbes, Dr. Penzance
Forbes, Dr. Vienna
Forbes, Dr. Chatham
Ford, Mr. Southsea
Forman, Mr. G. Ellery, Surg. London
Foster, Mr. W. Oakes, near Sheffield
Fothergill, Dr. W. Jamaica
Fosbrooke, Mr. Ross
Fodderick, George F. M. D. Dublin
Foote, Mr. Surgeon, Tavistock-street
Forster, Mr. Stafford-place
Foulkes, Edward, Esq. Surgeon, Eleven-towns
Fox, Mr. Surgeon, Huntingdon
Fowke, Mr. Surgeon, Wolverhampton
Fraser, Rev. Simon, Edinburgh
Freer, John, Esq. Chelsea
Freer, J. Esq. Assistant Surgeon 4th Light Dragoons
Franklin, H. Esq. Surgeon, Montreal
Freshfield, Mr. P. H. Surgeon, Oakley

G.

Gairdner, Dr. Dumfermline
Gallup, Mr. Joseph A. Woodstock, United States
Gaitskell, Mr. Surg. Rotherhithe
Gardener, Rev. F. Neston
Geddes, Mr. A. Noble, Surgeon
Gibbons, John, Esq. Ballymahon
Gibbs, Mr. Surg. Westbury, Wilts.
Gibney, Dr. Brighton
Gilbert, Dr. Theodore, Bermuda
Gilbert, Mr. James, Surgeon, Rochester
Gissing, G. E. Esq. Bury St. Edmunds
Goolden, R. Esq. Maidenhead
Gordon, Dr. Alexander, Finsbury-square
Gordon, Dr. Blenheim
Gordon, Mr. Oxford-street
Gorman, Mr. Surg. Manchester-street
Grabham, Mr. J. Surg. Rochefort
Graham, Dr. W. late of Jamaica
Graham, Mr. Oxford-court
Grant, Dr. Edinburgh
Grant, Mr. Surgeon, Bristol
Grattan, Dr. Dublin
Gray, Dr. R. N. Haslar
Grebke, Mr. R. N.
Green, Mr. W. Surg. Durham
Green, Dr. G. Yarum, Yorkshire
Green, Mr. Jos. Sep. Surgeon, Durham
Greenhead, Mr. late of Jamaica
Greenish, Mr. John, Surgeon, R. N. Sheerness
Grey, Sir Thomas, M. D.
Griffith, Mr. R. Flag-Surgeon, Mediterranean
Grimstone, E. Esq. Uxbridge
Gronous, Mr. West Indies
Gunning, Deputy-Inspector
Gunning, Mr. John, Surgeon to St. George's Hospital
Guthrie, J. G. Esq. Berkeley-st.
Guy, Mr. John, Surg. London

H.

Haden, Mr. Sloane-street
Hagley, Mr. Apoth. Oxford Infirmary
Halford, Sir H. bart. Curzon-st.
Hall, Mr. R. N.
Hall, Dr. John, Berwick
Halkard, E. Esq. Oldham, Lancashire
Hall, Mr. Robert, Durham
Hall, Mr. Grosvenor-st. Westm.
Hamilton, Dr. Finsbury-square
Hamet, Dr. East Indies
Hands, Mr. B. Turnham Green
Harding, Mr. Kentish Town
Harper, Mr. W. Manchester
Harshaw, Mr. Surgeon, R. N.
Harold, Philip, Esq. Surg. Holt, Norfolk
Harrison, Dr. R. Argyle-street
Harrold, J. P. Esq.
Hastings, Dr. Charles, Phys.

Hassel, Dr. E. Surg. Boulougne Sur. Mer
Hatfield, Mr. Thomas, Herts
Heenan Dr. Parson's-town
Henderson, Mr. Surgeon, Barton, Bristol
Hennen, Inspector, Edinburgh
Hendrickson, Mr. A. C. London
Higgs, Mr. John, Maidenhead, Berks
Higgins, Mr. F. Little Russell-street
Hill, Mr. D. Great Russell-street
Hillyar, Dr. Royal Navy
Hilliar, Mr. H. Dean-street, Borough
Highly, Mr. Elland
Hingeston, Mr. Y. Surg. Apoth. Coleman-street
Hudson, Mr. Surgeon, Lewes
Hogg, Mr. Littlehampton
Hollis, Mr. W. M. Surgeon
Holt, Mr. Surgeon, Tottenham
Holst, Dr. Public Health-officer, Norway
Hora, Mr. James, Surgeon, Harwich
Horsley, Dr. William, North Shields
Howel, Dr. Neath
Howell, Dr. Clifton
Howard, Dr. William, Baltimore
Howship, Mr. George-street
Hughes, Dr. Physician to the Forces, Barbadoes
Hull, Dr. Manchester
Hulbert, Mr. J. F. Surgeon, Melksham
Hume, Dr.
Hunt, Mr. Bombay
Hunter, Dr. Richmond
Hunter, Mr. Bishopsgate-street
Hunter, Dr. J. Lees, Witherby
Hunterian Society, St. Mary Axe
Huntley, Mr. L. Surg. Bristol
Hurst, Mr. James C. Surgeon, Dartford
Husband, Mr. W. Surg. York
Hutchison, Mr. A. C. London

I.

Illingworth, Mr. W. Surgeon, Hackney
Ives, Mr. Chertsey

J.

Jackson, Dr. Jamaica
Jardine, W. Esq. Surg. R. N. Dumfries
Jenner, Dr. Edward, Berkeley
Jenkins, Mr. Fossy, Surgeon, Sutton Valence
Jesson, Dr. Germany
Johnson, Edw. M.D. Weymouth
Johnson, D. Esq. Torrington, Devon
Johnston, Dr. Ireland
Johnston, Dr. Bengal Medical Establishment
Johnston, Andrew, Esq. Dublin
Johnston, Mr. K. Dowgate-hill
Jones, Dr. Dundalk
Jones, J. J. Esq. Margaret-street
Jones, Mr. Surg. Princes-street, Cavendish-square
Jones, Dr. George Haines, Portsmouth
Jordan, Mr. St. James's-square, Manchester
Jouenne, Dr. Rue de l'Orangerie, Brussels
Jubb, Mr. Hallifax
Junior, or Medico-Chirurgical Society, Portsmouth

K.

Keddell, Mr. Portsea
Kedgell, Mr. Richard, Surgeon, Wellington
Keenbyside, Mr. R. N.
Keith, Dr. James, Trinidad
Kennedy, Mr. C. Alverstone
Kennedy, Dr. Glasgow
Kenny, Dr. Castle Pollard
Kerr, Robert, Esq. Portobello, S. America
King, Mr. George, Surgeon, Harxest, Suffolk
King, Mr. G. Surg. H. C. Service Bengal
Kingsley, Dr. Templemore

Kingsley, Surg. Roscrea, Ireland
Kirby J. Esq. Dublin

L.

Lake, Mr. A. P. Surgeon, R. N. Teignmouth
Lamert, M. Esq. 1st Vet. Batt.
Lambert, Mr. Surg. Thursk
Lane, Mr. Arundel
Lanyon, Mr. R. Lostwithiel
Larver, Mr. 18th Regiment
Latham, Dr. John, Harley-street
Latham, Dr. P. M. Gower-street
Lawder, Surgeon
Lee, Dr. Rob. Melbourne-house, Whitehall
Leggett, Mr. Wm. St. Thomas's Hospital
Lempriere, Dr. Isle of Wight
Leslie, Mr. R. N.
Lewis, G. Esq. Surg. Sudbury
Lickorish, Mr.
Lillies, Mr. George, R. N.
Litchfield, Mr. Twickenham
Lithgow, Mr. Andrew, Surgeon, Weymouth
Little, Dr. Tandragee
Lloyd, Dr.
Lloyd, Mr. James, Ireland
Locock, Mr.
Lodge, Mr. Haymarket
Lucas, Dr. C. E. Hatfield
Luke, Dr. Argyle-street
Lutener, Mr. Bridgenorth
Lynn, Dr. Woodbridge
Lyne, Mr. Emsworth
Lynn Regis Medical-book Society
Lyon, Dr. Manchester
Lyster, Mr. 7th Dragoon Guards

M.

Macewan, Mr. George, Surgeon, Grenada
Madden, Mr. J. Malachi, R. N.
M'Alester, James, Esq. R. N.
M'Carthy, Dr. A. Skibbereen
Malcom, Dr. J. Perth
Magrath, George, M. D. F. R. S. and L. S. Plymouth
Mantell, Gideon, Esq. F. L. S. Castle-place, Lewes
Manchester Infirmary Committee
M'Arthur, Dr. Deal
Mardel, Mr.
Marshall, Mr. Assist. Surg. R.N.
Marshall, Mr. Surg. Belfast
Marks, Mr. W. Surg. Stapleton-place
Martin, Dr.
Mathews, Mr. Thomas, Leman, H. C. S. Prince Regent
Matthias, Mr. W. Gibraltar
Mauger, Mr. John, Surgeon, Guernsey
May, S. Esq. Ilfracombe
Maysmer, Mr. R. Surg. Ashbourne, Derbyshire
M'Calman, Mr. Duncan, Assist. Surgeon, Bengal
M'Carrogher, Dr. R. N.
M'Donnel, Dr. Belfast
M'Dougle, Dr. Paris
M'Donnel, J. (M.D.) 55 Regt. Infantry.
M'Donnell, Ephraim, Esq. Dublin
Medical Society, Chichester
Medical Society, Halifax
Medical Book Club, Edinburgh
Medical Society, Deal and Sandwich
Medical Society, Portsmouth
Medical Book Society, Kingston, Jamaica
Medico-Chirurgical Society, London
Medical Society, Bolt-court
Medical Association, Col. of Physicians, Dublin
Meek, Mr. Edinburgh
Merriman, Dr. Half Moon-street, Piccadilly
M'Grigor, Sir James, M. D.
Metcalfe, Mr. Thos Thompson, Sheffield
Miles, Mr. Surg. Throgmorton-st.
Miller, Mr. James, Perth
Millington, Mr. Titchfield-street
Milne, Dr. Edinburgh
Milne, Garden, M. D. Bamffe
Miln, Dr. Res. Phys. Naples

Mitchell, Mr. Keighley
Mitchel, Mr. Surgeon R. N.
Mitchel, J. Esq. Assist. Surg. 48th Regiment
Mackenzie, Dr. P. Great Ormond-street
M'Knight, Dr. W. G. Jamaica
M'Leod, Mr. John, R. N.
M'Leod, Dr. Opthalmic Hosp.
M'Millen, Mr. Surg. R. N.
Moffat, Mr. Panton-square
Moffatt, Mr. Surg. Long-lane
Money, Mr. Hanover-street
Monteath, Dr. G. C.
Montgomery, Mr. A. Surg. Paris
Moon, Mr. Medical Staff
Moore, Mr. Surg. Woodbridge
Moran, Francis, M. D. Ireland
Morel, Mr. New Bond-street
Morris, Mr.
Morrison, Dr. Newry
Morrison, W. Esq. 90th Light Infantry
Mortimer, Dr. Haslar
Morrison, Mr. W. Surgeon H.P. 12th Foot, Bristol
Mould, John, Esq. Surg. Oundle, Northamptonshire
Moule, Mr. W. B. Surg. 60th Reg.
Moulson, Dr. Hallifax
M'Rae, Mr. John, Surg. R. N. Old Broad-street
M'Ternan, P. Surgeon, R. N.
Mullins, Mr. John
Murray, Mr. John, Edinburgh
Musey, Dr. R. D. Dartmore College, U. S.
Muttlebury, Dr. Bath

N.

Nagle, Dr. R. N.
Napper, Mr. James, Surgeon, R. N. Paris
Nason, Dr. Ireland, (place not mentioned)
Neilson, Dr. Trinidad
Neilson, Dr. Dundalk
Newenham, Mr. W. Farnham
Newry Medical Society
Newsom, Mr. Surg. Rochester
Nicol, Dr. Sierra Leone
Nounan, Mr. Langport
Norwood, J. Esq. Ashford, Kent
North, Mr. Seymour-place
Nowood, Mr. Dover
Nunn, Mr. J. Surg. Holbrook
Nussey, Mr. St. James's-street
Nutt, James, Esq. R. N.

O.

O'Beirne, James, Esq.
Ogle, Richard, Esq. Great Russell-street
O'Malley, J. Esq. Surg. 11th Light Drag. Guards, Bengal
O'Meara, Mr.
O'Reilly, Richard, Esq. Dublin
Omodei, Signor, Milan
Osborne, Mr. R. N.
Osborne, Mr. Surg. Gracechurch-street
Outhwaite, Mr. Bradford

P.

Page, John, Esq. Dartmouth
Palmer, Dr. Tamworth
Paris, Dr. John A. Dover-street
Park, Mr. Surg. Kingsland-road
Parkinson, Mr. George, Racquet-court
Parmly, Mr. Surg. Dentist, Pall Mall
Parry, Dr. Charles, Bath
Pascalis, Felix, M. D. New York
Paxton, Sir William, Piccadilly
Peacock, George, Esq. Longford
Peebles, Surgeon, Ireland
Penrith Med. Society, Cornwall
Perkins, John, Esq. Brussels
Perkins, Mr. Measham
Percival, Dr. G. Bath
Pesket, Mr. East Aspling
Philip, Dr. A. P. W. Hanover-sq.
Phillips, Mr. Samuel, H. M. S. Nimrod
Peirce, Dr. Edward, Wexford
Pineo, Mr. R. N. Portsmouth
Pink, Mr. Portsea
Plowden, Dr. Midhurst, Sussex
Plumbe, Mr. Great Russell-street
Pomeroy, Dr. United States
Pool, John, Esq. Walton-upon-Thames

Porter, Mr. Portsea
Porter, Dr. Bristol
Porter, Dr. Arthur, New Hampshire
Porter, Mr. Surg. Bishopsgate-street
Powell, W. H. Esq. Manchester-street
Powell, Mr. Newman-street
Prevost, Dr. Switzerland
Price, Mr. W. United States
Price, Mr. W. Surgeon, R. N.
Probart, Mr. Surg. Hawarden
Proud, John, Esq. Surg. Wolverhampton
Punchon, Mr. Staff Surgeon
Pyne, Mr. W. Collard, Surgeon, Wellington
Pyper, Dr. 4th Dragoon Guards

Q.

Quarrier, Dr. R. M. A.
Quebec Medical Society

R.

Rankin, Dr. Marchmont-street
Reay, Mr. Surgeon
Reid, Robert, M. D. Dublin
Reilly, J. Esq. Plymstock
Richmond Hosp. Reading Room
Richie, Mr. Surg. Sierra Leone
Rickwood, W. Esq. Surgeon, Horsham
Rickwood, W. Jun. Horsham
Riley, E. Esq. Sydney, New South Wales
Ring, J. Esq. Hanover-st, Hanover-square
Risk, Dr. Saltash
Robertson, Dr. Northampton
Robertson, Dr. Edinburgh
Roberts, Mr. Manchester
Roberton, J. Esq. Hospital Assistant to the Forces
Robinson, Mr. Holles-street, Cavendish-square
Robinson, Mr. Mark, Beverly, Yorkshire
Robinson, Dr. N. J. Barry
Rodgers, Mr. J. Settle, Yorkshire
Rodd, Mr. Surgeon, Hampstead
Rodmel, J. Esq. Surg. R. N.
Rogers, Mr. H. M. S. Minden
Rogers, Mr. Worthing
Ross, Mr. Stirling
Roscoe, Mr. Richard
Rouzet, Dr. Rue de St. Pères à Paris
Royle, Mr. Sloane Street
Royal Institution, Albemarle-st.
Royal Ordnance Medical Society Woolwich
Royle, Mr. Surgeon, Chester
Rudge, Mr. Middlesex Hospital
Rudland, W. H. Esq. Surgeon, R. N. Dartmouth
Runciman, John, R. N.
Russell, Mr. Surg. Mount-street
Russel, Mr. G. J. London
Russell, Mr. R.
Rutherford, Dr.
Ryan, Dr. Ed. G. Kilkenny
Ryland, Mr.

S.

Sabine, Mr. Surg. Kington, Herefordshire
Sainsbury, W. M. D. Corsham, Wilts
Sanden, Dr. Chichester
Sanders, Dr. Edinburgh
Sankey, Mr. Dover
Sawyer, Mr. Margate
Scaife, Mr. Coxwould
Scatchard, Mr. J. Surgeon, near Leeds
Scalt, Mr. Surg. Esingwold, Yorkshire
Scott, Mr. Edinburgh
Scott, Dr. John
Scott, Mr. Surg. Easingwold
Scott, Dr. Russell-square
Scudamore, Dr. Wimpole-street
Seeds, Dr. Stafford, America
Settle, Joseph, Esq. Surgeon, Plymstock
Sexty, Mr. R. Surgeon, Hammersmith
Seymour, Mr. John, Gouldin, Hants
Sharpe, Mr. Bushire, Persia
Shaw, Mr. Surg. Albany, Piccad.
Shaw, Mr. Halifax

Shattuck, Dr. George P. United States
Shearman, Dr. W. Northampton-square
Shepherd, Mr. Witney
Sherwood, Ralph, Esq.
Shipton, Mr. Chipping
Shooldred, Mr. J. G. South Carolina
Short, Dr. P. M. O. St. Helena
Shoveller, Mr. R. N.
Shute, Mr. Surg. Gosport
Simpson, Mr. James, Edinburgh
Sims, Mr. Southsea
Simson, Mr. Knaresbro'
Skair, Mr. Castle-street, Edinburgh
Slow, Dr. Royal Horse Guards
Smalley, Mr. Robert, Richmond-buildings
Smart, Mr. Frith-street, Soho
Smith, Mr. Malta
Smith, Dr. J. Gordon, St. James's
Somerville, Dr. Hanover-square
Southey, Dr. Phys. to Middlesex Hospital
Sprague, J. H. Esq. Surgeon, Somerset
Sproule, Dr. Bombay
Squib, Mr. Saville Row
Stacy, Mr. London Hospital
Stanton, Dr. Moulton
Staunton, Mr. J. Longbridge
Stebbing, Mr. Ipswich
Steel, Dr. Walter, Edinburgh
Stedman, James, Esq. Guilford
Stenson, Dr. Burton-on-water, 2 copies
Steart, Augustus, W. Bath
Steel, Mr. Tower Hill
Stewart, Mr. Surg. St. James's-square, Manchester
Stewart, Mr. Edinburgh
Stewart, Mr. H. M. S. Bulwark
Stewart, Mr. Manchester
Stewart, Mr. H. M. S. Carron
Stewart, Mr. Southampton
Stevenson, Mr. M.R.C.S. Lond.
Stewart Dr. 71st Regt. Brussels
Stilon, Mr. Joseph, Malta
Stilwell, Mr. Walton-on-Thames
Stokoe, Dr. R. N.
Stolterforth, S. Esq. Bolton-st. Piccadilly
Stone, Mr. Thomas
Stowe, Mr. Surg. Buckingham
Strang, Mr. John, Surg. Bridport
Stuart, Dr. Bute, Wisbeach, Cambridgeshire
Sutcliffe, Mr. Rochedale
Swift, J. Esq. Surg. R. N,

T.

Tarn, Mr. J. Surgeon, H. M. S. Satellite
Taylor, Dr. David, Wotton-under-edge, Gloucestershire
Tedlie, Mr. Beresford, 85th Regiment
Tedlie, Dr. Cape Coast Castle, Africa
Thomas, Mr. Surg. Hatfield
Thomas, Mr. H. L. Leicester-square
Thompson, Dr. Belfast
Thompson, Dr. Paris
Thompson, Dr. Conduit-street
Thompson, Mr. Merchant, West Indies
Thornton, Dr. Edward, Stroud
Tickell, Mr. E. Burton, Somerset
Tobin, Mr. John, Surgeon
Todd, J. T. Esq. Havre de Grace
Todd, Dr. 83d Regiment
Todd, John, T. Esq. Pisa
Todd, C. H. Esq. Dublin
Travers, Mr. Bagholt
Travis and Dunn, Messrs. Scarborough
Trevasso, Mr. Surg. Falmouth
Turner, Mr. New-st, Baker-st
Twinton, Mr. Surg. Northwich

U.

Uwins, David, M. D. Bedford-row

V.

Vaile, W. Phelps, Esq. Surgeon, Headcorn, Kent
Valentine, Mr. John, London

Valentine, Mr. J. W. Surgeon, Sutton in Ashfield
Vaughan, Dr. Rochester
Vetch, John, M. D. F. R. S. E. Regent-street
Veitch, Dr. Pimlico
Vidal, Mr. Surg. Dean-st. Soho

W.

Wadd, Wm. Esq. Park-place, St. James's
Wake, Dr. Barth. Somerset
Walker, Dr. John, Walbrook
Wallis, Mr. Chas. Lincoln's-inn
Walker, Mr. J. E. Surg. Guilford-street
Walton, Mr.
Walker, Dr. Sanderson, St. Michael's, Azores
Walton, George, Esq. Surgeon E. I. C. Service
Wardrope, Mr. Arundel
Warder, Mr. R. N.
Wardrop, James, Esq. F.R.S.E. Charles-street
Warwick, Mr. R.N. Newfoundland
Warwick, Mr. R. Assist. Surg. R. N.
Watling, T. F. Esq.
Watson, S. Esq. Surg. Foot Gds
Weatherhead, Dr. Montague-st.
Webster, Mr. 51st Regt. L. I.
Webster, Mr. J. Leicester
Weir, Dr. Victualling-board
Wells, Silas, Esq. Burton-in-the-water
Whimper, Mr. G. M. Surgeon, Tillingham
Whiteing, The Rev. Mr. G. B. Hitching, Herts
White, Mr. B. at Mr. Brookes's
White, W. R. Esq. 84th Regiment
Whitney, Mr. 85th Regiment
Whitter, Dr. Bridge-street, Blackfriars
Wight, Dr. Robt. Madras
Wight, Mr. Chertsey
Williamson, Mr. Whitehaven
Williamson, Dr. Whitehaven
Wilmore, Edmund W. Surgeon, Worcester
Wilmore, Mr. John, Surgeon, Worcester
Wilson, Mr. Surgeon, Belfast
Wilson, J. Esq. F. R. S. George-street
Williams, Mr. Wm. Llandovery
Willis, Mr. Ratcliffe Highway
Williams, Mr. G. Portsea
Williams, Dr. W. H. Ipswich
Wilson, Mr. Surgeon, Arbroath, North Britain
Williamson, James, Esq.
Wilkinson, Mr. Richard, Dudley, Staffordshire
Willson, Sir Alex. M. D. Mead House
Willsher, Mr. G. Weathersfield, Essex
Winborne Medical Society, Dorsetshire
Wood, Sir James Athol, Albany, Piccadilly
Wood, Mr. Surgeon, Middleton, York
Woodcock, Mr. J. Surg. Bury, Lancashire
Woodman, Mr. R. Surg. Weymouth
Woodyer, C. Esq. Guilford
Worcester Med. & Surg. Society
Wright, Mr. George-street, Portman-square

Y.

Yates, Dr. T. Brighton
Yonge, Dr. Henrietta-st. Cavendish-square
York Hospital Library
Young, Mr. W. Surg. Ballymena
Young, Dr. James Forbes, Paradise-place
Young, Dr.

☞ Some of the Subscribers of this Quarter, are not inserted in the general list. They will be inserted in the next Volume. Full half of the actual Purchasers of the Journal are yet unknown. We can only insert those Names that are transmitted to us.

### INDEX.

A.

Abercrombie, Dr. on enteritis.. 161
——————— on consumptive diseases .................... 767
Abuse and use of mercury...... 41
Acid, Muriatic, in worm cases.. 89
Adams, Sir W. on artificial pupil 71
Alcock, Mr. on mucous mem-
——————— on the palate.... 457
branes ...................... 341
Aloes, Dr. Paris's remarks on.. 89
American medical science, review of..................... 512
Ammonia caustic, its effects.... 472
Ancle-Joint, dislocations of, &c. 60
Anderson's pills, composition of 89
Aneurisms, Mr. Pattison on.... 757
Animals, how different from man 238
Annuaire medico-chirurgicale.. 59
Anthrax of the penis.......... 30
Anus artificial, cure of........ 518
Aorta obliterated.............. 507
Apoplectic puerperal convulsions 132
Apoplexy, Dr. Pearson on...... 320
——————— Drs. Cooke, Abercrombie, Bricheteau, Rochoux, and Serres, on ................ 1
Apothecaries' resolutions ...... 325
Apothecaries, letters to........ 204
Army medical board, its zeal .. 299
Arnott, Mr. on the urethra..... 743
Arsenic, Dr. Paris's test for.... 89
Atheism ...................... 417

B.

Bailey, Dr. on fungoid tumour.. 477
Baillie, Dr. on paraplegia...... 391
Ballingall, Dr. on syphilis...... 581
Bampfield, Mr. on entero-eplocele ........................ 484
Barclay's anti-bilious pills .... 90
Bark, in constipation of the bowels ...................... 226
Bell, Mr. Charles, on the urethra 493
——————— on the urethra, &c.. 264
Beer, Professor, on artificial pupil 75
Bingham, Mr. on strictures.... 544
Blanc, Sir G. on vaccination.... 718
Blood, transfusion of.......... 117
Blood-letting in difficult parturition ............ 233
——————— in epidemic fever,, 143
Blood-letting in puerperal convulsions ................. 134
Blundell, Dr. on transfusion of blood ..................... 117
Books reviewed in Vol. I.—
1. Drs. Cooke, Abercrombie, Bricheteau, Rochoux, and Serres, on apoplexy, 1—2. Evans on genital ulcerations, 28—3. Hamilton on mercurial medicines, 41—4. Depuytren on fractures, 59.—5. Guthrie and Adams on artificial pupil, p. 71—6. Med. Chir. Trans. v. ix. 98, 104, 109, 117—7. Hutchinson on tic douloureux, 111—8. Millingen on military hygiene, 125—Luscombe on ditto, 125—9. Dr. Dewees on puerperal convulsions, 128 — 10. Drs. Grattan and Crampton on fever. 139—11. Weatherhead on rickets, 153 — 12. Drs. Broussais, Gasc, Abercrombie, and Pemberton, on peritoneal inflammation, 161—13. Dr. Hall on puerperal affection, p. 195—14. Letters on the practice of physic and surgery, 204—15. Mr. White on strictures of the rectum, 208 — 16. Mr. Howship on diseases of the intestines, 222—17. Dr. Dewees on difficult parturition, 229—18. Drs. Haslam, Burrows, and Esquirol on insanity, 237—19. Messrs. Bell and Shaw on the urethra, 264—20. Dr. Hennen on military surgery, 283—21. Mr. Cross on varioloid diseases, 300—22. Dr. Hastings on bronchitis—23. Dr. Prichard on fever, 381 — 24. Transactions of the College of Physicians, vol. vi. p. 387—25. Calcutta reports on cholera, 409—26. Mr. Pring on the laws of organic life, 416—27. Mr. Wilson's lectures on the bones, 427—28. Dr. Gregory's practice of physic, 435—30. Dr. Hutchinson on infanticide, 444—31. Supplemental review, 452—32. Retrospective review of American medical science, 512—33. Dr. Merriman on difficult labours, 526—34. Mr. Bingham on strictures, 544—35. Brera on worms, 565—36. Dr. Ballingall on syphilis, 581—37. Sir B. Faulkner on the plague, 586—38. Dr. Burns on midwifery, 595—40. Drs. Scudamore and Mackenzie on mineral waters, 605—41. Transactions of the College, vol. vi. p. 622—42. Dr. Elliotson on prussic acid, 655—43. Dr. M'Cabe on Cheltenham waters, 665—44. Dr. Vetch on diseases of the eye, 668—45. Mr. Whiter on the disorder of death, 687—46. Dr. Forbes on the climate of Penzance, 695—47. Dr. Davies on Mercury, 700—48. Dr Granville on prussic acid, 702—49. Dr. Cooke on nervous diseases, 725—50. Mr. Arnott on the dilator, 743
Bones, not affected by mercury 47
Bowels, constipation of........ 227
Brera, Professor, on worms.... 565
Bricheteau. Dr. on Apoplexy .. 1
Bronchitis, Dr. Hastings on.... 353
Broussais on chronic inflammation ......... 161 341
——— cases of peritonitis 187
Burns, Mr. on midwifery ...... 595
Burrows, Dr. on insanity...... 237

C.

Calculous diathesis, Dr. Philip on........................ 621
Carter, Dr. on warm climates.. 396
Caries of bones, by mercury.... 285
Catheter, how to be used...... 270
Caustic bougies condemned.... 270
Cephalea, relieved by solanum 401
Cerebral apoplexy ............ 20
Chancres, difficult of discrimination .............. 286
———, how to be treated.... 286
———, different modifications of................ 288
Chicken-pox, Mr. Cross on.... 304
Chevelier, Mr. on relaxed rectum ....................... 481
Cheltenham waters, Dr. M'Cabe on......................... 665
Cholmeley, Dr. case of malformation ..................... 395
Cholera, epidemic, of India.... 409
Chronic peritonitis............. 169
Circular, from the Army Medical Board, respecting syphilis .. 292
Circulation, Dr. Lucas on...... 329
Coley, Mr. on the rectum...... 218
Colchicum, seeds of .......... 319
Colles, Dr. on lymphatic glands 122
——— on polypous tumours 544
College transactions, vol. vi.... 387
Constipation of the bowels .... 226
——— in general .. 250
Consumptive diseases ........ 767
Cooke, Dr. on nervous diseases 1
Cow-pox, Mr. Cross on........ 302
Copaiba in bronchitis ......... 375
Corbyn, Mr. on cholera........ 409
Cooke, Dr. on Palsy .......... 725
Colchicum, Dr. Williams on.... 769
Coxarius morbus, cure of...... 521
Crampton, Dr. on fever........ 145
Crane, Dr. on pyrola umbellata 320
Croup, Treatment of.......... 57
Cutaneous diseases, new cure for 506
Cynanche laryngea, dreadful case of ..................... 416

D.

Dalby's carminative .......... 94
Dandelion, decoction of ......... 97
Davies, Dr. on mercury........ 700
Davy, Dr. on cholera........... 415
Dean, Dr. on the ear ......... 455
Death, disorder of............. 687
Delivery, a particular disease of, 195
Dementia, picture of.......... 249
Delivery in puerperal convulsions ................... 135
Deity, existence of, denied .... 418
Dewees, Dr. on puerperal convulsions............. 128
——— on difficult parturition............ 229
Diarrhœa, peculiar species of .. 56
Dickson, Dr. on peritonitis 182, 166
——— on erysipelas.... 761
Dilatation (artificial) of os uteri 598
Dinner pills.................. 89
Dislocations of the ancle-joint 60
Diuretics, observations on .... 84
Douglas, Dr. on hour-glass contraction ................ 647
Dropsy, Dr. Hamilton on...... 56
Dropsy in bronchitis.......... 378
Drowning, curious theory of... 450
Ducamp, Dr. on fever ........ 383
Duodenum, peculiar disease of.. 51
——— Dr. Yeats on...... 639
Dupuytren, M. on the ancle-joint 60
Dysmenorrhœa, Dr. Dewees on 495

E.
Earle, Mr. on deafness ........ 452
Elaterium and elatin........... 90
Elliotson, Dr on prussic acid .. 655
Empyema, Baron Larrey on.... 462
Epidemic fever in Ireland...... 139
Ergot in difficult labour ...... 236
Eruptive diseases, pathology of 345
Epidemic small-pox at Norwich 300
Erysipelas, Mr. Evans on...... 34
——— Dr. Dickson on .... 761
Esquirol, Dr. on insanity...... 237
Evans, Mr. on ulcerations of the penis ..................... 27
Excoriation, Mr. Evans on.... 33
Eye, Dr. Vetch on diseases of.. 668

F.
Farr, Mr. on scrofula.......... 120
Faulkner, Sir Brooke on plague 586
Femur, neck of, fractured...... 498
Fever, Dr. Grattan on........ 139
——— Dr. Crampton on...... 145
——— Dr. Prichard on........ 381
Fistula in ano................. 281
Fits, Dr. Latham on.......... 629
Fœtus, size and weight of...... 446
——— spontaneous evolution of 627
——— in utero, affected by syphilis .............. 47
Forbes, Dr. on Penzance...... 695
Forceps, (obstetric) remarks on 601
Fractures of the fibula ........ 60
Fungoid tumour, Dr. Bailey .... 477

G.
Gardiner, Dr. disease of the heart 461
Gase on peritoneal inflammation 161
Gastrotomy, two cases of...... 101
——— on Apoplexy .... 25
Genital organs, diseases of.... 28
Gooch, Dr. on spontaneous evolution ............ 627
——— on puerperal insanity 631
Granville, Dr. ovario-gestation 485
——— on prussic acid.. 702
——— reply to reviewer 803
Grattan, Dr. on fever.......... 139
Gregory, Dr. M.S. lectures.... 4
——— on enteritis...... 167
——— practice of physic 435
Guthrie, Mr. on artificial pupil.. 71

H.
Herpes preputialis............ 31
Hamilton, Dr. on mercury .... 41
Hutchinson, Mr. on tic douloureux........................ 111
Hunterian Society............ 157
Hydatid origin of tubercles.... 171
Hall, Dr. on peritonitis........ 182
——— on puerperal affections 195
Halford, Sir Henry, on prognosis 650
Halloran, Dr. on insanity in Ireland ...................... 242
Harrogate waters ............ 611
Harrison, Dr. on mucous membranes ................... 341
Haslam, Dr. on insanity...... 237
Hastings, Dr. on bronchitis.... 341
Hæmorrhoids, Mr. Bell on .... 280
Heart, remarkable disease of.. . 458
——— Dr Gardiner on......... 461
——— disease of ............ 771
Hennen, Dr. on military surgery 283
——— on syphilis ...... 284
——— on hydrothorax .. 474
Hereditary organization in insanity ..................... 254
Hernia, cold in .............. 389
Hernia with hæmorrhage...... 488
Hooping cough, Dr. Robertson on 770
Hour-glass contraction of the uterus ................... 647
Howship, Mr. on peritonitis.... 183
——— on the lower intestines......... 222
Hutchinson, Dr. on infanticide . 444
Hydrothorax, Dr. Hennen on.. 474
Hydrocyanic acid, Dr. Elliotson on......................... 655

I. J.
Idiotism, picture of............ 250
Incontinence of urine, cure for.. 266
Infant, *in utero*, infected ...... 47
Inflammation simulated ...... 123
Inflammation and irritation.... 195
Intestines, Mr. Howship on.... 222
Insanity, Drs. Haslam, Burrows, and Esquirol on............ 237
Insanity, a corporeal disease.. 239
Insanity, curability of ........ 256
——— pathology of ........ 257
——— treatment of ........ 258
Injection of the bladder........ 267
Intestines, painful affections of.. 402
Infanticide, Dr. Hutchinson on 444
Intestines, wounds of.......... 489
Inflammation of arteries...... 502
Insanity puerperal, Dr. Gooch on 631
Inflammation peritoneal ...... 161
Iritis, after the non-mercurial treatment of syphilis......... 294
James's analeptic pills ........ 96
Jameson, Mr. on cholera...... 409
Jenner, Dr. on vaccination .... 718
——— circular .......... 780
Johnson, Dr. reply to reviewers 551

K.
Kerchoffs, Dr. medical observations....................... 394

L.

Labour protracted, remarks on 604
Latham, Dr. on venesection.... 629
Latham, Dr. on solanum tuber. 400
Larrey Baron, on empyema.... 462
——— on wounded intestines.... 469
Lectures on the skeleton, Mr. Wilson's.... 427
Legal medicine, uncertainty of.. 450
Life, Mr. Pring on the laws of.. 416
Liq. morphii citratis.... 127
—— ant. tart. Dr. Paris on.... 92
Luscombe, Dr. on hygiene.... 125
Luxations of the ancle joint.... 60
Lymphatic glands, Dr. Colles on 122

M.

M'Sweeny, Dr. spicula of bone.. 480
M'Cabe, Dr. on Cheltenham waters.... 665
Malta, plague of.... 586
Masturbation, a cause of insanity.... 252
Materialism, effects of.... 252
Mackenzie, Dr. on mineral waters.... 605
Matlock waters.... 609
Matthias, Mr. case of hernia.... 488
Man, how different from animals 238
Marks, Mr. Case of tetanus.... 158
Materia Medica, vicissitudes of 81
Meningeal apoplexy.... 19
Mercury, Dr. Hamilton on.... 41
——— necessary in true syphilis.... 46
Mercury, Dr. Davies on.... 700
Mercury, good effects of in syphilis.... 285
Melancholia, picture of.... 249
Menstruation suppressed.... 107
Medico-chirurgical annals.... 59
Mercury, operation of, promoted 85
Melancholia cured by mercury.. 508
Merriman, Dr. on difficult labours 526
Medical transactions, vol. vi... 387
Membranes, adventitious.... 404
Medical Board, on cholera.... 409
Meatus auditorius, disease of.... 452
Muriat of lime, Dr. Hamilton on 53
Mercury in croup,.... 57
Military hygiene.... 125
Millengen, Dr. on hygiene.... 125
Midwifery, Mr. Burns on.... 595
Mineral waters, work on.... 605
Montfalcon on peritonitis.... 161
Monomania, picture of.... 249
Modified small-pox.... 311
Mucous membrane of the lungs 341
Mott, Dr. on ligature of arteria innominata.... 562

N.

Nephritis calculosa, case of.... 109
Neck of the bladder inflamed.. 268
Neuralgia, M. Vaidy on.... 764
Nitro-muriatic acid bath.... 53
Norwich, variolous epidemic at 300

O.

Ophthalmia, observations on.. 100
Opium in difficult parturition.. 233
——— hurtful in insanity.... 263
Ovario-gestation, case of.... 485
Ovarian dropsy.... 57
Oxymur. hyd. Dr. Paris on.... 91

P.

Paralysis, its peculiar connexion with apoplexy.... 16
Parturition difficult, Dr. Dewees on.... 229
——— Dr. Merriman on 526
Painful affection of the intestines 402
Paraplegia, Dr. Baillie on.... 391
Passions, causes of insanity.... 253
Paris, Dr. on pharmacologia.... 80
Palsy, Dr. Cooke on.... 725
Pattison, Mr. on aneurisms.... 757
Paris, Dr. pharmacologia, 4th ed. 550
Palate, congenital division of.. 456
Pearson, Dr. on apoplexy.... 320
Peritoneal inflammation, essay on 161
Pemberton, Dr. on the abdominal viscera.... 161
Peritonitis chronica.... 169
——— pathology of.... 172
——— acute, treatment of.. 177
——— chronic, treatment of 185
——— cases of.... 187
Penzance, climate of.... 695
Pharmacologia, Dr. Paris on.... 80
Phlegmon of the penis.... 29
Physic, practice of, Dr. Gregory 435
Philip, Dr. on calculous diathesis 623
Plumbi superacetas, virtues of.. 94
Plague, Mr. Webb on.... 407
Placenta over the os uteri.... 522
Plague, Sir B. Faulkener on.... 586
Powell, Dr. on the intestines.. 402
Poisoning by verdigrease.... 158
Post mortem changes in the body 122
Porter, Dr. on a preparation of opium.... 127
Prichard, Dr. on fever.... 381
Prostate gland, Mr. Bell on.... 264
Prolapsus ani, Mr. Bell on.... 279
Practice of physic and surgery in England.... 204
Prophylaxis in apoplexy.... 27
Pring, Mr. on organic life.... 416
Prognosis, Sir H. Halford on.. 650

Pring, Mr. broaches atheistical principles .................. 417
Prussic acid, Dr. Elliotson on.. 555
Prussic acid, Dr. Granville on.. 702
Psoriasis preputialis.......... 31
Ptyalism, remarkable case of .. 45
Pupil artificial, Mr. Guthrie on 71
——— Sir W. Adams on 71
——— Professor Bëer on 75
Purgative drink, elegant form of 86
Puerperal affections, Dr. Hall on 195
Puerperal convulsions, Dr. Dewees on.................... 128
Pulmonary consumption, Dr. Carter on.................. 396
Pyrola umbellata, Dr. Crane on 320

Q.

Quack medicines, composition of 89

R.

Reasoning, curious specimen of 420
Reform among apothecaries.... 204
Researches on apoplexy......... 1
Religion, a cause of insanity.... 243
Rectum, Mr. Bell on.......... 264
Retention of urine............ 269
Rectum, Mr. Chevalier on .... 481
——— Mr. White on the.... 208
Reviewers, infallibility of...... 445
Rickets, Dr. Weatherhead on .. 153
Rigby, Dr. his humanity...... 302
Rickets, Mr. Wilson on........ 428
Rochoux, Dr. on apoplexy .... 1
Romero, Dr. on hydro-pericardium ..................... 477
Robertson Dr. on hooping cough 770

S.

Scott, Mr. his academic institution ...................... 158
Scrofula, Mr. Farr on ........ 119
Scudamore, Dr. on mineral waters ...................... 605
Scirrhous contraction of the rectum.................... 278
Serres, Dr. on apoplexy......... 1
Sexual intercourse, effects of .. 33
Separation from friends in insanity ...................... 259
Sensibility, morbid, of the bladder ...................... 266
Shaw, Mr. Editor of Mr. Bell's work ..................... 264
Sherril, Dr. on disease of the heart ................... 762
Skeleton, lectures on........... 427
Skull, bony tumour of ........ 98
Solanum tuberosum ........... 400
Sodaic powders, composition of 96
Spine, distortions of.......... 431
Stimulants in apoplexy........ 26
Stricture of the rectum, Mr. Bell on.................... 275
Strictures, Mr. Bingham on.... 544
Stanley, Mr. on superfœtation.. 654
Staphyloma, Dr. Vetch on .... 674
Strictures made by bougies .... 265
Stomach, affections of ......... 50
Strains of the ancle-joint...... 61
Strictures of the rectum, Mr. White on.................. 208
Suicide not prevalent in England 242
Supplemental reviews.. 98, 452, 757
Syphilis, Dr. Hamilton's remarks on.................. 46
——— Dr. Hennen on...... 283
——— non-mercurial treatment of.......... 284
——— letter from the Army Board............ 292
——— in fœtus in utero.... 298
——— Dr. Ballingall on.... 581
Sympathetic pains in the urethra 272

T.

Tabes mesenterica............. 500
Tar vapour in bronchitis ...... 372
Tetanus cured by bleeding, &c. 158
Thoracic affections, ammonia in, 472
Thigh, luxation of............ 500
Tic douloureux, Mr. Hutchinson on......................... 111
Toxicological chart .......... 337
Transfusion of blood.......... 117
Tracheotomy, singular case of.. 416
Tunbridge wells............... 610
Tubercle of the penis ......... 80

U.

Ulcerations of the penis ...... 28
Urinary calculi, Dr. Paris on. 32
Urethra, Mr. Bell on.......... 264
——— on the anatomy of .... 265
——— sympathies of ........ 265
——— Mr. Arnott on ........ 743
Urine, suppression of ........ 269
——— retention of, relief of .. 519
Uterine hæmorrhage ......... 486
——— hæmorrhage........... 153
Uterus, extirpation of ........ 104
——— rupture of............ 108

V.

Variolous, epidemic, at Norwich 300
Varioloid diseases ............ 304
Vaccination, its degree of protection.................... 300
——— Sir G. Blane on.... 718
——— Dr. Jenner on .... 718
Varicella, history of .......... 309
Vaidy, M. on thoracic affections 472
Vesica urinaria Mr. Bell, on.... 264

Venerola vulgaris ............ 34
———— superficialis ........ 38
———— indurata............ 38
Venereal poison in the blood .. 47
Vetch, Dr. on the curability of insanity ........ 241
———— on diseases of the eye 668
Verminous diseases .......... 565
Venesection, Dr Latham on .. 629
Vomits in apoplexy .......... 25

W.

Wardrop, Mr. on irritability.... 501
Warts within the rectum...... 274
Webb, Mr. on plague ........ 407
Weatherhead, Dr. on rickets .. 153
White, Mr. on strictures of the rectum.................. 208
Whiter, Mr. on disorder of death 687
Widows of medical officers .... 157
Williams, Dr. on colchicum.... 319
———————— on ditto........ 769
Williams, Mr. on uterine hæmorrhage ................ 155
Wilson's lectures on the skeleton 427
Worms, Professor Brera on.... 565
Wounds of the chest.......... 462

Y.

Yeats, Dr. on cold in hernia .. 389
Yeats, Dr. on the duodenum .. 639

---

P. S. We have to regret that several Subscribers' names, and alterations of titles, arrived after the Journal was closed, and when we had no other place to mention the circumstance than this. It is particularly requested that Subscribers will send their names before the 10th of the Publication month in each quarter.

☞ We are requested by Sir James M'Grigor to say, that the venerable and able Dr. Jackson, after a tour of the Ionian Isles, Constantinople, Gibraltar, Cadiz, &c. for the purpose of investigating the subjects of Plague and Fevers of those places, has returned, and is putting to press the result of his investigations. We can have no doubt that our brethren will shew their respect for this worthy member of the profession, by their patronage of the work, *viz.* before the 10th *May*, 10th *August*, 10th *November*, and 10th *February*.

---

*Directions to the Binder.*

The Tables of Contents are to be taken from the four Numbers published, and to be bound up with the Volume after the Title Page. They are paged for that purpose. The Plate in No. 1, is to face page 68—that in No. 3, to face page 456—and that in No. 4, to face page 756.

